# Investigating Relationships Among Self-Efficacy, Mood, and Anxiety Using Digital Technologies: Randomized Controlled Trial

**DOI:** 10.2196/45749

**Published:** 2023-08-14

**Authors:** Judith Rohde, Marta Anna Marciniak, Mirka Henninger, Stephanie Homan, Christina Paersch, Stephan T Egger, Erich Seifritz, Adam D Brown, Birgit Kleim

**Affiliations:** 1 Department of Psychiatry, Psychotherapy and Psychosomatic Psychiatric University Hospital Zurich Zurich Switzerland; 2 Department of Psychology University of Zurich Zurich Switzerland; 3 University Hospital of Child and Adolescent Psychiatry and Psychotherapy, University of Bern Bern Switzerland; 4 Department of Psychology New School for Social Research New York, NY United States; 5 Department of Psychiatry New York University School of Medicine New York, NY United States

**Keywords:** self-efficacy, digital intervention, digital assessment, EMA, EMI, mood, anxiety, emotional flexibility

## Abstract

**Background:**

Digital tools assessing momentary parameters and offering interventions in people’s daily lives play an increasingly important role in mental health research and treatment. Ecological momentary assessment (EMA) makes it possible to assess transient mental health states and their parameters. Ecological momentary interventions (EMIs) offer mental health interventions that fit well into individuals’ daily lives and routines. Self-efficacy is a transdiagnostic construct that is commonly associated with positive mental health outcomes.

**Objective:**

The aim of our study assessing mood, specific self-efficacy, and other parameters using EMA was 2-fold. First, we wanted to determine the effects of daily assessed moods and dissatisfaction with social contacts as well as the effects of baseline variables, such as depression, on specific self-efficacy in the training group (TG). Second, we aimed to explore which variables influenced both groups’ positive and negative moods during the 7-day study period.

**Methods:**

In this randomized controlled trial, we applied digital self-efficacy training (EMI) to 93 university students with elevated self-reported stress levels and daily collected different parameters, such as mood, dissatisfaction with social contacts, and specific self-efficacy, using EMA. Participants were randomized to either the TG, where they completed the self-efficacy training combined with EMA, or the control group, where they completed EMA only.

**Results:**

In total, 93 university students participated in the trial. Positive momentary mood was associated with higher specific self-efficacy in the evening of the same day (*b*=0.15, SE 0.05, *P*=.005). Higher self-efficacy at baseline was associated with reduced negative mood during study participation (*b*=–0.61, SE 0.30, *P*=.04), while we could not determine an effect on positive mood. Baseline depression severity was significantly associated with lower specific self-efficacy over the week of the training (*b*=–0.92, SE 0.35, *P*=.004). Associations between higher baseline anxiety with higher mean negative mood (state anxiety: *b*=0.78, SE 0.38, *P*=.04; trait anxiety: *b*=0.73, SE 0.33, *P*=.03) and lower mean positive mood (*b*=–0.64, SE 0.28, *P*=.02) during study participation were found. Emotional flexibility was significantly enhanced in the TG. Additionally, dissatisfaction with social contacts was associated with both a decreased positive mood (*b*=–0.56, SE 0.15, *P*<.001) and an increased negative mood (*b*=0.45, SE 0.12, *P*<.001).

**Conclusions:**

This study showed several significant associations between mood and self-efficacy as well as those between mood and anxiety in students with elevated stress levels, for example, suggesting that improving mood in people with low mood could enhance the effects of digital self-efficacy training. In addition, engaging in 1-week self-efficacy training was associated with increased emotional flexibility. Future work is needed to replicate and investigate the training’s effects in other groups and settings.

**Trial Registration:**

ClinicalTrials.gov NCT05617248; https://clinicaltrials.gov/study/NCT05617248

## Introduction

Technological development and the ubiquitous presence of smartphones entail that digital assessments and interventions gain presence and importance in mental health. These novel digital methods show great potential to advance mental health research, and there is sound evidence for their feasibility and high compliance [1]. Ecological momentary assessments (EMAs) are a smartphone-based method to measure fluctuating and transient parameters such as mood, activity, and within-person dynamics, avoiding retrospective bias and supporting longitudinal and ecological validity [2]. Ecological momentary interventions (EMIs) provide an efficient way of digitally delivering psychological interventions, both disorder-specific and transdiagnostic, that fit well into individuals’ daily lives and routines [3]. EMIs offer convenient mental health support that is scalable, resource-saving, and satisfying for users [4]. Furthermore, they seem to increase their efficacy when combined with an experience-based sampling methodology (eg, EMA) [3].

Self-efficacy refers to a person’s belief in their ability to succeed in a particular situation and is commonly associated with positive mental health outcomes [5,6]. It positively affects people’s motivation and ability to manage personal, social, professional [5,6], and academic challenges [7,8]. Self-efficacy can be circumscribed in 2 aspects. First, general self-efficacy, which is situation-independent and refers to an individual’s competence in dealing with challenges; and second, specific self-efficacy, which is situation-dependent and relates to an individual’s belief to perform well in a specific field [9-11]. Self-efficacy beliefs have been identified as protective factors, for example, against psychological distress in times of uncertainty and adversity, such as during a pandemic [12-14] and counteract depressive episodes in students [15].

A growing body of research has shown that perceptions of self-efficacy can be experimentally manipulated (eg, via false feedback techniques), which may result in clinically relevant improvements in cognitive, affective, and decision-making processes [16-18]. Furthermore, perceptions of self-efficacy can be increased by recalling autobiographical mastery experiences. These strategies are based on Bandura’s concept that self-efficacy beliefs are construed partly from the internalization of successfully overcoming difficult life episodes [10]. For example, the induction of mastery-related autobiographical memories outperformed the induction of positive autobiographical episodes in reducing distress and subjective physiological responses [19]. Other studies applied this memory-based self-efficacy induction to clinical populations. They found, for example, that the capacity to recall autobiographical self-efficacy memories was linked to better social problem-solving and goal-oriented future thinking [20].

Based on these findings, we developed an EMI providing a 1-week digital, that is, smartphone-based, self-efficacy training based on the recall of autobiographical self-efficacy memories. We combined this training with EMA of mood, specific self-efficacy, the social context of the current situation, and web-based contact with others. In this study, situation-dependent specific self-efficacy was assessed in the training group (TG) only and refers to the participants’ belief in completing and benefiting from the study’s tasks. The investigation of the effects of the training on the outcomes of self-efficacy, anxiety, hopelessness, perceived stress, positive and negative affect, and hope was part of a different manuscript. We found significant reductions in anxiety and hopelessness from baseline to postintervention and a significant increase in self-efficacy when controlling for baseline self-efficacy [21].

This study focuses on the daily measures we captured using EMA. Based on previously found associations between mood and self-efficacy, we hypothesized that the daily captured mood was associated with the daily captured specific self-efficacy. Additionally, we wanted to explore how the other daily captured mental health–related aspects and baseline depression and anxiety interacted with mood and specific self-efficacy, aiming to gain a more nuanced picture of the effects of the training and to investigate relationships between these parameters.

## Methods

### Study Design and Participants

**Figure 1:**
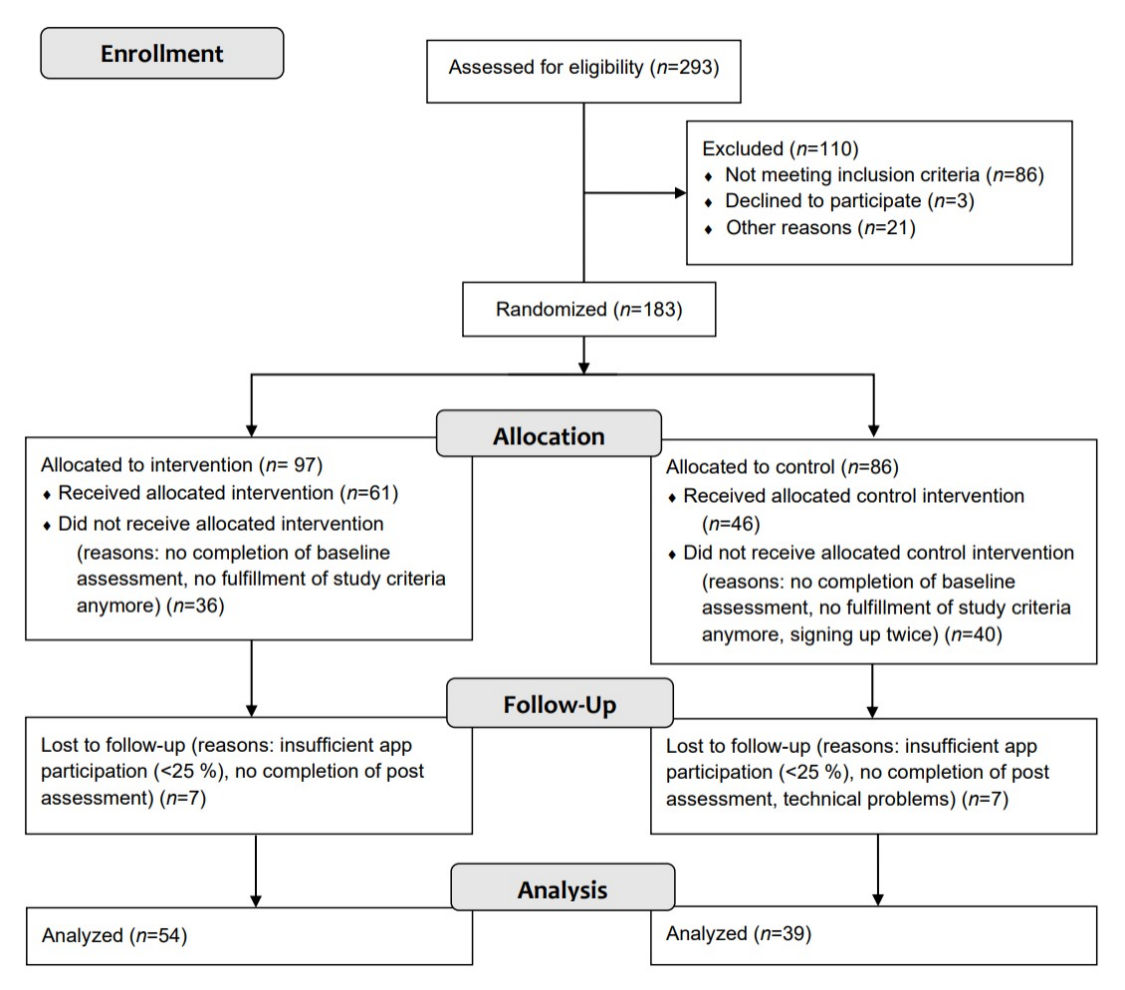
CONSORT (Consolidated Standards of Reporting Trials) flow diagram.

We conducted a randomized controlled trial with 93 student participants who had reported elevated levels of stress (score of ≥13 on the Perceived Stress Scale [PSS] [22,23]). Because of an expected dropout rate of about 50% [24], we recruited 183 participants who were randomly assigned to a digital autobiographical self-efficacy memory training and EMA (TG) or EMA only (control group [CG]). Independent researchers conducted random allocations. Which group participants were assigned to was blinded. During study participation, we excluded participants who did not fulfill inclusion criteria anymore at study start, who did not complete the baseline or postassessment, or who did not sufficiently participate in the app, which was defined as responding to at least 25% of the prompts. The final sample consisted of 93 participants (n=54 in the TG and n=39 in the CG; Figure 1). For a detailed description of the methods, please see our prior publication [21].

### Recruitment

Participants were recruited from universities in Switzerland using mailing lists and postings on university websites and social media. Participants received detailed information about the study, its procedures, duration, potential risks, and benefits, but no specific information on the different groups. For example, they were informed about a 1-week smartphone app that assessed thoughts and emotions daily, which applied to both groups and secured blinding. All participants provided informed consent. They were offered monetary compensation, depending on compliance or course credits.

### Screening

Interested students were asked to complete a web-based screening questionnaire assessing basic demographic information and their current stress level using the PSS. Inclusion criteria were (1) being a student at a Swiss university, (2) being 18-29 years old, (3) being a smartphone owner, (4) having fluent German language skills, and (5) having a score of ≥13 on the PSS. Exclusion criteria were a current psychiatric disorder.

### Procedures

In this study, we used 2 apps. One providing EMA and EMI (TG) and one providing EMA only (CG). Consecutively, both groups received EMA [25] 10 times per day, of which 3 were combined with the self-efficacy training in the TG. Besides, the TG received 8 additional EMA questions on specific self-efficacy every evening. EMA was adapted from Marciniak and colleagues [26]. As described in various studies using EMI and EMA, we also allowed participants to self-trigger interventions and assessments [4]. EMA questions assessed current mood, social and web-based contacts in the current situation, and specific self-efficacy (in TG only).

In addition to the scheduled prompts, participants could trigger additional EMA and EMI (TG), respectively, and EMA (CG) at any time. Both apps provided general information about study procedures and app use, and the TG app also offered a self-efficacy review section. EMI and the EMA were administered through the SEMA^3^ (Melbourne eResearch Group) platform, an open-source software application for delivering advanced smartphone surveys [27]. Participants were able to download the app from the app store or by using a link they received via email.

Before the app participation, participants in the TG watched video-based psychoeducation on self-efficacy [28] and were instructed to define 2 autobiographical self-efficacy memories. Specifically, they were asked about difficult life episodes they have overcome. The self-efficacy training consisted of instructions to slow down breathing and to perform an imagination task, specifically to recall their autobiographical memories step-by-step. For example, participants were instructed to initially build up contextual factors and, ultimately, to focus on the skills and character traits relevant to success [25]. After every training, we asked for the participant’s feedback on how well it went.

### Ethics Approval

The trial obtained ethical approval from the Ethics Committee of the Faculty of Arts of the University of Zurich (20.4.24). Since this is not a clinical trial (ie, only healthy participants were involved), preregistration is not mandatory. The trial was registered retrospectively (ClinicalTrials.gov NCT05617248). The authors assert that all procedures contributing to this work comply with the ethical standards of the relevant national and institutional committees on human experimentation and the Helsinki Declaration, as revised in 2013.

### Measures and Outcomes

#### Baseline and Post Assessment

Participants completed web-based baseline and post assessments (within 1 week after the last app use), which consisted of the German versions of the Beck Depression Inventory-II (BDI-II; [29,30]), the State-Trait Anxiety Inventory (STAI; [31,32]), the Positive and Negative Affect Scale (PANAS; [33,34]), the Beck Hopelessness Scale [35,36], the General Self-Efficacy Scale (GSE; [37,38]), and the Trait Hope Scale [39,40]. The post assessment also included the PSS and the Mobile Application Rating Scale, User Version [41,42].

#### Daily Measures Using EMA

Using EMA, we measured mood, social contacts, and web-based contacts 10 times per day (EMA questions are publicly available via Open Science Framework; see note 2). The first 10 questions focused on mood and were rated on a 7-point Likert scale (1=not at all to 7=very much). We measured positive mood using 3 items (cheerful, happy, and relaxed) and negative mood using 7 items (irritated, anxious, insecure, lonely, sad, overthinking, and stressed). The following section explores social contacts. Two options indicated dissatisfaction with social contacts: (1) being with nobody and feeling excluded or wanting to be with someone; or (2) being with someone instead of wanting to be alone. We combined these into 1 variable. The last section of the EMA focused on web-based contacts. We asked who the person was in web-based contact with at that moment and how they felt about it.

The TG received 8 additional EMA questions on specific self-efficacy every evening. These questions did not measure general self-efficacy but specific situation-dependent self-efficacy, which referred to participation in the self-efficacy trainings. For example, participants were asked about their level of agreement with statements such as “Right now, I think I will be able to do what is necessary to make this training successful.”

#### Primary, Secondary, and Additional Outcomes

The analysis focuses on the daily EMA, which captures mental health aspects such as mood, satisfaction with social and web-based contacts, and specific self-efficacy. In addition, we investigated how these variables collected as additional outcomes interacted with each other and with baseline variables. The investigation of the changes in the primary (self-efficacy) and secondary outcomes (stress, positive and negative affect, hope, anxiety, and hopelessness) was part of a different manuscript [21].

### Statistical Analyses

#### Sample Size and Power Analysis

Power analysis was conducted in RStudio using the *sjstats* package. For α=.05, power 80%, and an effect size of *d*=0.6, the required sample size was 94 participants, which is in line with our recent review summarizing findings from mobile health EMI studies [4].

#### Data Analyses

Descriptive statistics (percentages, means, and SDs) were used to present the baseline characteristics of the sample. We present our findings in text, tables, and figures. We applied a type I error rate of α=.05 in all analyses.

First, we were interested in whether we could predict self-efficacy through mood and other EMA variables as well as through baseline variables. For this purpose, we used multilevel modeling for repeated measures to assess which variables influenced specific self-efficacy during the self-efficacy training in the TG. With measurement occasions on level 1 nested in participants on level 2, we assessed the effects of mood and dissatisfaction with social contacts on specific self-efficacy during the self-efficacy training within (level 1) and the effects of baseline variables on specific self-efficacy during the training between (level 2) participants.

Second, we used a multivariate multilevel model to assess the effects of the experimental group (EMA and EMI vs EMA only) and dissatisfaction with social contacts on positive and negative mood, including autoregressive mood effects. As before, level 1 was defined by the measurement occasions and level 2 by participants (see note 2 for access to complete data tables and codes).

All analyses were conducted in R (version 4.0.2; R Core Team) and Mplus (version 8.6) [43] using the MplusAutomation package (Hallquist and Wiley) and SPSS (version 27.0; IBM Corp).

## Results

### Sample Characteristics

The total sample consisted of 93 (54 in the TG and 39 in the CG) participants. The mean age was 23.72 (SE 0.44) years in the TG and 22.64 (SE 0.61) years in the CG (group difference: *P*=.14). There were 42 female participants in the TG and 31 female participants in the CG (group difference: *P*=.84). Table 1 summarizes clinical variables. There were no statistically significant differences between groups in demographic and baseline psychological variables. Participants reported no harm or unintended effects.

**Table 1:** Clinical variables and group differences.

Baseline psychological variables		Training group (n*=*54)		Control group (n*=*39)			Group difference
		Mean	SE	Mean	SE	*P* value^a^	
Perceived Stress Scale		22.48	0.81	21.28	0.95	.34	
Beck Depression Inventory		13.96	1.23	11.64	1.43	.22	
**State and Trait Anxiety Scale**							
	State anxiety	43.65	1.57	42.41	1.73	.60	
	Trait anxiety	45.41	1.58	41.67	1.45	.085	
**Positive and negative affect schedule**							
	Positive affect	26.35	1.01	27.05	1.20	.66	
	Negative affect	16.93	0.93	16.74	0.89	.89	
	Beck Hopelessness Scale	5.57	0.63	4.15	0.56	.11	
	General Self-Efficacy Scale	26.94	0.57	27.90	0.73	.30	

^a^Independent samples *t* test for continuous data.

### Predicting Specific Self-Efficacy in the TG

**Figure 2:**
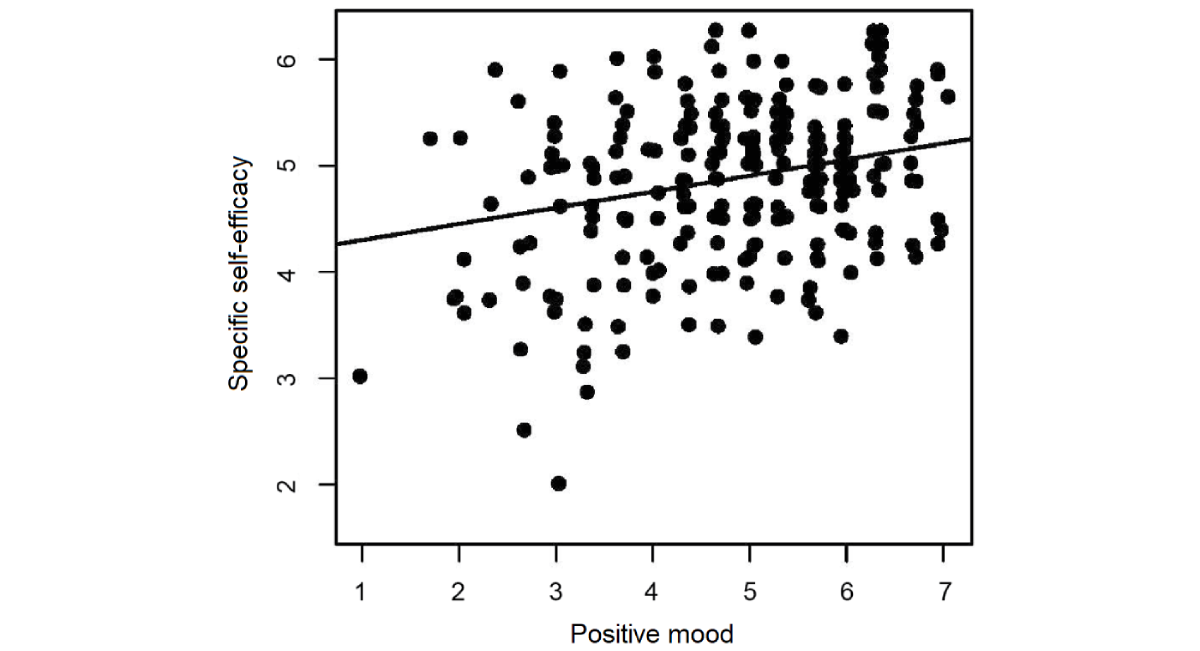
Association of positive mood and specific self-efficacy. The association between specific self-efficacy and positive mood was significant (*P*<.01).

Within participants in the TG, positive mood during the day was associated with higher specific self-efficacy in the evening (*b*=0.15, SE 0.05, *P*=.005; Figure 2). Neither negative mood during the day, dissatisfaction with social contacts, nor specific self-efficacy on the previous day had any significant effect on specific self-efficacy.

**Figure 3:**
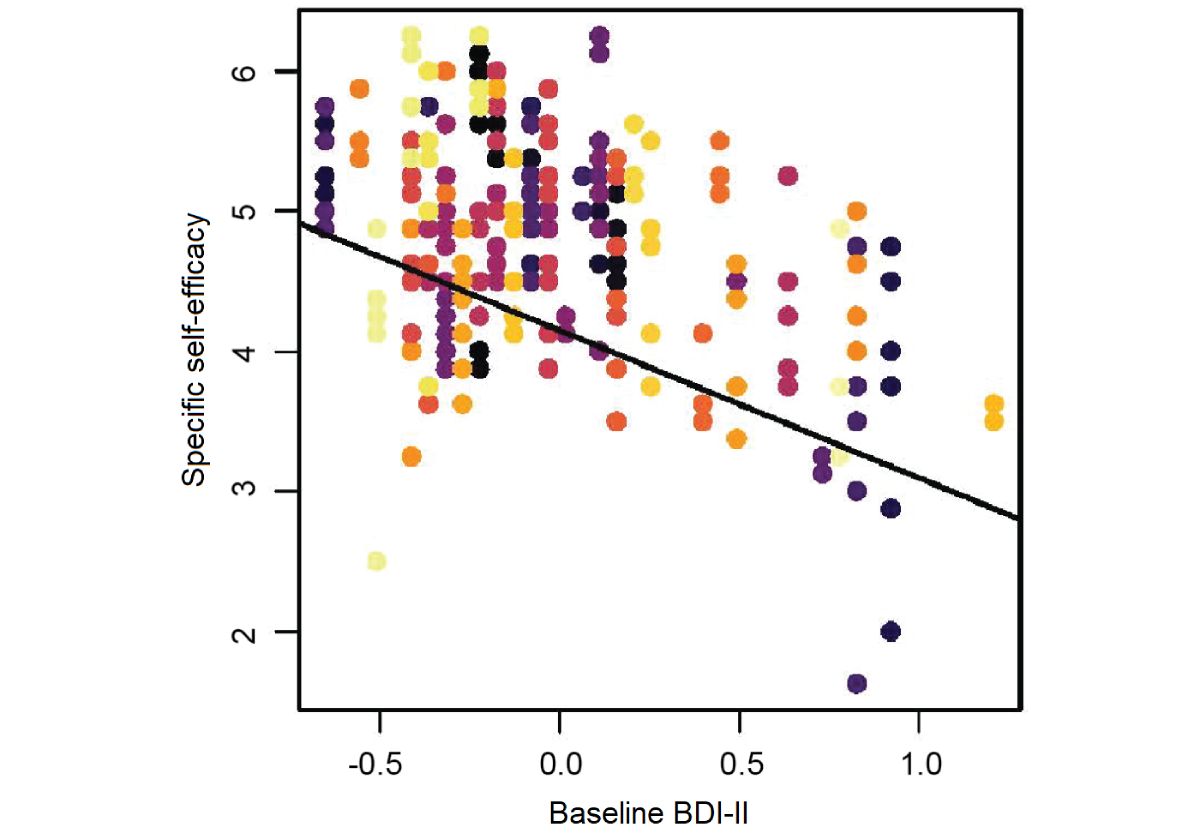
Association of baseline depression (BDI-II) with specific self-efficacy throughout the self-efficacy training. Each participant’s different levels of specific self-efficacy in the course of the training are colored using the same color; the association between baseline depression and specific self-efficacy was significant (*P*<.01). BDI-II: Beck Depression Inventory-II.

Between participants, more severe baseline depressive symptoms (BDI-II) were associated with lower specific self-efficacy (mean score over 1 week) during the self-efficacy training (*b*=–0.92, SE 0.35, *P*=.004, Figure 3). Other baseline variables showed no significant effect on specific self-efficacy. For detailed results, see Table 2.

**Table 2:** Predicting specific self-efficacy in the training group: results of the multilevel model.

Outcome and predictor		Estimate	SE	Z value	*P* value	Within-person or between-person effects
**Specific self-efficacy**						
	Positive mood^a^	0.15	0.05	2.82	.005	Within
	Negative mood^b^	–0.002	0.08	–0.03	098	Within
	Dissatisfaction with social contacts^c^	–0.12	0.12	–0.96	.34	Within
	Day^d^	–0.02	0.05	–0.44	.66	Within
	Day/self-efficacy (GSE^e^)	–0.14	0.11	–1.30	.19	Within
**Specific self-efficacy**						
	Perceived stress (PSS^f^)	–0.19	0.21	–0.91	.37	Between
	Depression (BDI-II^g^)	–0.92	0.35	–2.65	.008	Between
	State anxiety (STAI State^h^)	0.10	0.28	0.37	.71	Between
	Trait anxiety (STAI Trait^i^)	0.43	0.26	1.65	.10	Between
	Positive affect (PANAS Pos^j^)	–0.11	0.15	–0.74	.46	Between
	Negative affect (PANAS Neg^k^)	0.03	0.18	0.17	.87	Between
	Self-efficacy (GSE)	0.45	0.27	1.68	.09	Between

^a^Positive mood: EMA items “cheerful,” “happy,” “relaxed.”

^b^Negative mood: EMA items “irritated,” “anxious,” “insecure,” “lonely,” “sad,” “overthinking,” “stressed.”

^c^Dissatisfaction with social contacts: EMA items either “being with nobody” + “feeling excluded” / “rather wanting to be with someone” or “being with someone” + “rather wanting to be alone.”

^d^Day: log-linear time trend.

^e^GSE: General Self-Efficacy Scale.

^f^PSS: Perceived Stress Scale.

^g^BDI-II: Beck Depression Inventory-II.

^h^STAI State: State-Trait Anxiety Inventory (state subscale).

^i^STAI Trait: State-Trait Anxiety Inventory (trait subscale).

^j^PANAS Pos: Positive and Negative Affect Schedule (positive affect subscale).

^k^PANAS Neg: Positive and Negative Affect Schedule (negative affect subscale).

### Daily Mood During Study Participation

Within participants, we found autoregressive effects for positive (*b*=0.34, SE 0.04, *P*<.001) and negative (*b*=0.48, SE 0.05, *P<.*001) mood in both groups. The effect of a positive mood on the following day’s mood was independent of group allocation. In contrast, the effect of negative mood was reduced in the TG (*b=*–0.13, SE 0.05, *P=*.01). Dissatisfaction with social contacts was associated with both a decreased positive mood (*b*=–0.56, SE 0.15, *P<.*001) and an increased negative mood (*b=0*.45, SE 0.12, *P<.*001) in both groups, with no significant group difference.

Between participants, trait anxiety at baseline (STAI, trait) was associated with lower positive mood (*b*=–0.64, SE 0.28, *P*=.02), while positive affect at baseline was associated with increased positive mood during study participation (*b*=0.34, SE 0.12, *P*=.004). More depressive symptoms at baseline (BDI-II) were associated with reduced negative mood (*b=*–1.05, SE 0.47, *P*=.02), while high state and trait anxiety (STAI, state and trait) were associated with increased negative mood (state anxiety: *b*=0.78, SE 0.38, *P*=.04; trait anxiety: *b*=0.73, SE 0.33, *P*=.03). Higher self-efficacy at baseline (GSE) was associated with reduced negative mood during study participation (*b*=–0.61, SE 0.30, *P*=.04); we observed no effect on positive mood. The other variables, including group membership, showed no significant effect on mood. For detailed results, see Tables 3 and 4.

**Table 3:** Daily mood during study participation: results of the multivariate multilevel model for within-person effects.

Outcome and predictor		Estimate	SE		Z value		*P* value
**Positive mood**							
	Positive mood^a^	0.34	0.04	8.57		<.001	
	Negative mood^b^	–0.15	0.04	–3.92		<.001	
	Dissatisfaction with social contacts^c^	–0.56	0.15	–3.70		<.001	
	Day^d^	–0.03	0.041	–0.65		.52	
	Group/mood	–0.07	0.04	–1.65		.099	
	Group/dissatisfaction with social contacts	–0.17	0.20	–0.86		.39	
	Day/self-efficacy (GSE^e^)	–0.14	0.12	–1.19		.23	
**Negative mood**							
	Negative mood	0.48	0.05	10.03		<.001	
	Positive mood	–0.04	0.02	–2.39		.02	
	Dissatisfaction with social contacts	0.45	0.12	3.67		<.001	
	Day	0.020	0.03	0.58		.56	
	Group/mood	–0.13	0.05	–2.49		.01	
	Group/dissatisfaction with social contacts	0.08	0.15	0.56		.58	
	Day/self-efficacy (GSE)	0.07	0.11	0.66		.51	

^a^Positive mood: EMA items “cheerful,” “happy,” “relaxed.”

^b^Negative mood: EMA items “irritated,” “anxious,” “insecure,” “lonely,” “sad,” “overthinking,” “stressed.”

^c^Dissatisfaction with social contacts: EMA items either “being with nobody” + “feeling excluded” / “rather wanting to be with someone” or “being with someone” + “rather wanting to be alone.”

^d^Day: log-linear time trend.

^e^GSE: General Self-Efficacy Scale; within-person correlation between positive and negative mood was –.352.

**Table 4:** Daily mood during the study participation: results of the multivariate multilevel model for between-person effects.

Outcome and predictor		Estimate	SE	Z value	*P* value
**Positive mood^a^**					
	Group	0.06	0.14	0.43	.66
	Perceived stress (PSS^b^)	0.001	0.30	0.003	.10
	Depression (BDI-II^c^)	0.37	0.41	0.90	.37
	State anxiety (STAI State^d^)	–0.23	0.34	–0.68	.50
	Trait anxiety (STAI Trait^e^)	–0.64	0.28	–2.28	.02
	Positive affect (PANAS Pos^f^)	0.34	0.12	2.86	.004
	Negative affect (PANAS Neg^g^)	0.16	0.21	0.74	.46
	Self-efficacy (GSE^h^)	0.52	0.28	1.86	.06
	Group / perceived stress (PSS)	0.39	0.37	1.06	.29
	Group / depression (BDI-II)	–0.92	0.52	–1.77	.08
	Group / state anxiety	–0.51	0.43	–1.20	.23
	Group / trait anxiety	0.39	0.38	1.02	.31
	Group / positive affect (PANAS Pos)	–0.25	0.24	–1.06	.29
	Group / negative affect (PANAS Neg)	–0.06	0.32	–0.20	.84
	Group / self-efficacy (GSE)	–0.87	0.54	–1.61	.11
**Negative mood^i^**					
	Group	–0.02	0.15	–0.14	.89
	Perceived stress (PSS)	0.23	0.24	0.97	.33
	Depression	–1.05	0.47	–2.25	.02
	State anxiety	0.78	0.38	2.07	.04
	Trait anxiety	0.73	0.33	2.21	.03
	Positive affect (PANAS Pos)	0.06	0.24	0.26	.80
	Negative affect (PANAS Neg)	0.54	0.37	1.47	.14
	Self-efficacy (GSE)	–0.61	0.30	–2.08	.04
	Group / perceived stress (PSS)	–0.12	0.34	–0.35	.73
	Group / depression (BDI-II)	0.54	0.61	0.88	.38
	Group / state anxiety (STAI State)	–0.77	0.50	–1.55	.12
	Group / trait anxiety (STAI Trait)	0.41	0.43	0.94	.35
	Group / positive affect (PANAS Pos)	0.24	0.33	0.73	.47
	Group / negative affect (PANAS Neg)	–0.11	0.46	–0.25	.80
	Group / self-efficacy (GSE)	0.19	0.59	0.32	.75

^a^Positive Mood: EMA items “cheerful,” “happy,” “relaxed.”

^b^PSS: Perceived Stress Scale.

^c^BDI-II: Beck Depression Inventory-II.

^d^STAI State: State-Trait Anxiety Inventory (state subscale).

^e^STAI Trait: State-Trait Anxiety Inventory (trait subscale).

^f^PANAS Pos: Positive and Negative Affect Schedule (positive affects subscale).

^g^PANAS Neg: Positive and Negative Affect Schedule (negative affects subscale); between-person correlation between positive and negative mood was –0.228.

^h^GSE: General Self-Efficacy Scale.

^i^Negative Mood: EMA items “irritated,” “anxious,” “insecure,” “lonely,” “sad,” “overthinking,” “stressed.”

## Discussion

### Principal Results

This study applied an EMI providing a 1-week digital self-efficacy training to university students reporting elevated stress levels. We analyzed the daily collected EMA data and investigated the prediction of specific self-efficacy and how mood and other momentary parameters interacted with each other and with baseline variables. Positive training effects have been reported elsewhere [21].

We found several associations between mood and self-efficacy in university students with elevated stress levels: (1) a positive association between momentary positive mood and specific self-efficacy in the evening of the same day, (2) an association between higher baseline self-efficacy and lower mean negative mood during study participation, and (3) an association between higher baseline depressiveness and lower mean specific self-efficacy during study participation. Additionally, we found associations between anxiety and mood: (1) higher baseline trait and state anxiety were associated with a higher mean negative mood during study participation, and (2) higher baseline trait anxiety was associated with a lower mean positive mood during study participation. Moreover, we found autoregressive mood effects in both groups, but the autoregressive effect of negative mood was reduced in the TG.

### Comparison With Prior Work

Our results confirm previous studies that show an association between positive mood and self-efficacy [6,19]. The relation between high baseline depressiveness and low specific self-efficacy during study participation may be due to the negative association between depression and motivation, interests, and cognitive performance [44-46]. This finding also matches previous results suggesting that negative mood in depressed people is associated with a lower capacity to learn from new experiences [47], which may impede engagement in and understanding of the self-efficacy training. Interestingly, it has been shown that specific self-efficacy moderates the relationship between mood and cognitive performance, as it positively affects cognitive performance, but only during a positive mood [48].

The relationship between anxiety and mood is in line with literature widely investigating and discussing that depression and anxiety share some aspects and are correlated [49]. Within participants, positive mood and negative mood were self-reinforcing, a common effect of inertia, and in line with the assumption that such effects are implicated specifically in individuals with psychological maladjustment, low self-esteem, depression, stress, and rumination [50-53]. Interestingly, the self-reinforcing effect of positive mood was independent of group membership, while the self-reinforcing effect of negative mood was alleviated by self-efficacy training.

The training may enhance mood and enable participants to manage difficulties better when faced with challenging situations. The training may also have contributed to higher emotional flexibility [50], which is particularly important for adaptation and coping in constantly changing environments [54,55] and is associated with well-being, resilience [56], emotional functioning, and quality of life [57]. Interestingly and complementary to our results, a recent pilot study showed improvements in general self-efficacy in a group that had received emotional flexibility training [58].

Specific self-efficacy correlates with self-efficacy [59,60]. Previous research has shown that the specificity of self-efficacy plays an important role within a domain and even within a specific task [61,62]. Future investigations could go deeper and, for instance, assess specific self-efficacy concerning the content of the self-efficacy memories and, if applied in different settings, such as before or in addition to specific therapies, participants’ therapy motivation, therapeutic relation, and other parameters related to the specific situation, since self-efficacy has been associated with improved therapy motivation [63], treatment adherence, and outcomes [64,65].

### Limitations and Strengths

This study is not without limitations. First, the part of our analyses this study focuses on was exploratory. Aiming to gain more knowledge on specific self-efficacy and a more nuanced picture of the potential effects of the self-efficacy training, we examined the prediction of specific self-efficacy in the TG and associations of mood with other EMA and baseline parameters in addition to our main research questions focusing on the effects of the training, which we described in another publication [21]. Such an approach amplifies the probability of false-positive findings.

The study was conducted during the second wave of the COVID-19 pandemic, and we included participants with elevated stress levels detected by using the PSS, which measures the degree to which life has been perceived as unpredictable, uncontrollable, and overwhelming [22,23]. Thus, we intended to include a subpopulation that is at higher risk since psychological stress is, for example, associated with negative consequences for academic performance and with mental disorders in students [66-68]. However, we did not investigate if or to what extent the elevated stress levels were related to the pandemic. Due to the pandemic, college students may have had to change their lifestyles, which may have had negative consequences on their mental health [69]. However, other stressors are arguable, as college students experience a wide range of distressing factors related to the university environment and its challenges and responsibilities [70]. Additionally, we cannot preclude that other active ingredients besides the self-efficacy training might have contributed to the observed effects. Additionally, the option to self-trigger EMI and EMA leads to less random EMA data collection, which might bias the results. Other limitations are the homogeneity of our sample, for example, regarding education, age, and origin; the unequal group sizes; and the reduced generalizability since we investigated a sample with elevated stress. Furthermore, the study is slightly underpowered because our final sample consisted of 93 participants instead of the intended 94. Therefore, replications with larger, more heterogeneous samples and in different settings are warranted.

Our study has several strengths. First, we showed multiple associations between mood and self-efficacy, respectively, specific self-efficacy leading to the conclusion that participants with lower baseline depression scores benefit more from the training than participants with higher baseline depression scores, giving the practical implication that enhancing self-efficacy might be more effective if individuals additionally receive training on how to elicit positive emotions or learn antidepressant strategies. The self-efficacy training showed a positive effect on the emotional flexibility needed to counterbalance the carryover effects of low mood.

EMA data is extremely powerful in describing and explaining momentary symptoms and momentary symptom fluctuations. In this study, we focused on the within- and between-person relationships between various symptom measures, mood, and self-efficacy. It would be an interesting avenue for future research to also explore the time-varying effects of how the constructs impact each other.

### Conclusions

This study showed several significant associations between mood and self-efficacy as well as between mood and anxiety in students with elevated stress levels. Improving mood in participants with low mood could enhance the effects of self-efficacy training. The self-efficacy training could be especially beneficial for individuals with low emotional flexibility and contribute to mental health improvements. Additionally, analyses could focus on time-varying effects in which the constructs impact each other. Learning more about when and in which time frames changes in symptoms, mood, and self-efficacy occur might support practitioners in developing new psychological interventions.

## Appendix

Multimedia Appendix 1 CONSORT-eHEALTH checklist (V 1.6.1).

## Abbreviations

BDI-II: Beck Depression Inventory-II
CG: control group
EMA: ecological momentary assessment
EMI: ecological momentary intervention
GSE: General Self-Efficacy Scale
PANAS: Positive and Negative Affect Scale
PSS: Perceived Stress Scale
STAI: State-Trait Anxiety Inventory
TG: training group

## Data Availability

The data sets analyzed during this study and the codes are available and can be accessed at [25]. Detailed results are included in this study.
